# Development and Electromyographic Validation of a Compliant Human-Robot Interaction Controller for Cooperative and Personalized Neurorehabilitation

**DOI:** 10.3389/fnbot.2021.734130

**Published:** 2022-01-18

**Authors:** Stefano Dalla Gasperina, Valeria Longatelli, Francesco Braghin, Alessandra Pedrocchi, Marta Gandolla

**Affiliations:** ^1^NeuroEngineering and Medical Robotics Laboratory (NearLab), Department of Electronics, Information and Bioengineering, Politecnico di Milan, Milan, Italy; ^2^WE-COBOT Lab, Polo Territoriale di Lecco, Politecnico di Milano, Lecco, Italy; ^3^Department of Mechanical Engineering, Politecnico di Milan, Milan, Italy

**Keywords:** neurorehabilitation, human robotics, compliant control, impedance control, electromyography, physical human-robot interaction

## Abstract

**Background:**

Appropriate training modalities for post-stroke upper-limb rehabilitation are key features for effective recovery after the acute event. This study presents a cooperative control framework that promotes compliant motion and implements a variety of high-level rehabilitation modalities with a unified low-level explicit impedance control law. The core idea is that we can change the haptic behavior perceived by a human when interacting with the rehabilitation robot by tuning three impedance control parameters.

**Methods:**

The presented control law is based on an impedance controller with direct torque measurement, provided with positive-feedback compensation terms for disturbances rejection and gravity compensation. We developed an elbow flexion-extension experimental setup as a platform to validate the performance of the proposed controller to promote the desired high-level behavior. The controller was first characterized through experimental trials regarding joint transparency, torque, and impedance tracking accuracy. Then, to validate if the controller could effectively render different physical human-robot interaction according to the selected rehabilitation modalities, we conducted tests on 14 healthy volunteers and measured their muscular voluntary effort through surface electromyography (sEMG). The experiments consisted of one degree-of-freedom elbow flexion/extension movements, executed under six high-level modalities, characterized by different levels of (i) corrective assistance, (ii) weight counterbalance assistance, and (iii) resistance.

**Results:**

The unified controller demonstrated suitability to promote good transparency and render both compliant and stiff behavior at the joint. We demonstrated through electromyographic monitoring that a proper combination of stiffness, damping, and weight assistance could induce different user participation levels, render different physical human-robot interaction, and potentially promote different rehabilitation training modalities.

**Conclusion:**

We proved that the proposed control framework could render a wide variety of physical human-robot interaction, helping the user to accomplish the task while exploiting physiological muscular activation patterns. The reported results confirmed that the control scheme could induce different levels of the subject's participation, potentially applicable to the clinical practice to adapt the rehabilitation treatment to the subject's progress. Further investigation is needed to validate the presented approach to neurological patients.

## 1. Introduction

Worldwide, stroke is a leading cause of death and permanent disability (Johnson et al., 2016). Although the global mortality of stroke has decreased in the past decades, the incidence and the effects of the disease are expected to increase (Gorelick, 2019). Consequently, the burden of stroke is still likely to produce long-term impairment, limitations during activities of daily living, and compromise the social participation of most stroke survivors. In most cases, rehabilitation treatment is required for an effective recovery, besides partial spontaneous recovery. Indeed, physical therapy fosters the motor relearning process during post-stroke rehabilitation. Nevertheless, only 5–20% of people with initial upper limb impairment after stroke completely recover lost functionalities (French et al., 2016). In the past years, the literature proposed upper limb robot-assisted rehabilitation as a method to stimulate motor relearning through repetitive, high-intensity, and task-oriented functional training (Winstein et al., 2016; Duret et al., 2019). Since the 90s, several upper-limb robotic devices have been designed, but only a few of them effectively reached the market, probably due to controversial results obtained in clinical trials (Ambrosini et al., 2019).

Recent systematic reviews show that robotic rehabilitation could produce better, or at least equivalent, outcomes than conventional therapy in both the International Classification of Functioning, Disability and Health (ICF) Body and Activity domains (Veerbeek et al., 2017; Mehrholz et al., 2018, 2020). Moreover, given that traditional passive mobilization limits neuroplasticity, a more customizable and adaptable control approach, facilitating subject's engagement and motivation, could lead to better effectiveness of the treatment (Marchal-Crespo and Reinkensmeyer, 2009). Thus, the effectiveness of the robotic rehabilitation therapy strongly depends on the capability of the system to guide natural coordinated motion, promote physiological muscular contraction, and induce the patient to cooperate as much as possible. This is why a key component of effective robot-mediated therapy is a good cooperative and adaptable control solution, which can be tailored to the single user being able to follow his/her progress.

With this study, we first analyze the robot-mediated rehabilitation modalities proposed in the literature. We investigate the availability of low-level control strategies that can be exploited to promote the desired haptics and physical human-robot interaction. Finally, we present the description of a low-level unified controller for upper-limb rehabilitation that is capable of assisting patients in a compliant manner and that promotes most of the robot-mediated training modalities used in clinics.

The fundamental concept of the proposed approach relies on the availability of a unified compliant controller, which could change the level of assistance and resistance according to the patient's performances and contribution, toward the paradigms of personalization and continuity of care. The core idea is that by tuning three control parameters, we can change the perceived haptics of the test-bed when interacting with the human arm. We validated this concept by monitoring surface EMG while asking (healthy) subjects to modulate their volitional contribution to correctly fulfill the required task.

### 1.1. Structure of the Study

This study is structured as follows. Section 2 defines the rehabilitation training modalities used in upper-limb robot-assisted therapy and their low-level control implementation challenges. The core idea of this study is presented in section 3, which explains how high-level modalities have been integrated with a low-level unified compliant controller. Section 4 presents the experimental design implemented to test the controller, and the results are exposed in section 5. Finally, sections 6 and 7 draw the discussion and conclusion of the Chapter.

## 2. Related Study and Novel Contribution

### 2.1. Robot-Mediated Rehabilitation

Robot-mediated rehabilitation has been largely investigated since the 1990s. The literature agrees that the goal of robots should be to induce motor plasticity in subjects undergoing rehabilitation treatment and, therefore, to improve their motor recovery (Huang and Krakauer, 2009). Therefore, robot-mediated control algorithms were designed and developed, taking inspiration from motor learning and neurophysiological aspects (Krakauer, 2006; Reinkensmeyer et al., 2016; Iandolo et al., 2019). Consequently, different high-level training modalities were proposed to promote motor recovery at different stages of the disease. Such modalities are in turn embodied by low-level controllers that are capable of shaping the physical human-robot interaction (pHRI) according to the residual capabilities of the user, i.e., the aim of researchers is to design controllers that minimize the interaction forces between the robot and the human while motivating the subject and guaranteeing the completion of the rehabilitation task. In other words, the robot should cooperate with the patient along with the rehabilitation treatment as a therapist would do, changing the levels of assistance, resistance, and motion correction based on the progression of the motor recovery. Over the past decade, several reviews on exoskeletal control for robot-assisted rehabilitation have been proposed in the literature. However, the researchers proposed several taxonomies and categorizations at various levels of abstraction (Marchal-Crespo and Reinkensmeyer, 2009; Basteris et al., 2014; Meng et al., 2015; Gull et al., 2020). In this study, we will use the term “training modalities” for “high-level” desired rehabilitation behaviors and the term “control strategies” for their “low-level” control scheme implementation. Generally, the training modalities for upper-limb rehabilitation are characterized by three main features: (i) corrective assistance, which implies that, given a pre-defined task, the system also corrects the movement when the subject moves away from the desired trajectory; (ii) weight counterbalance assistance, which refers to the ability of the robot to support and compensate the weight and the dynamics of the impaired limb; and (iii) resistance, which relates to training strategies that make the movement more difficult to perform, thus engaging the subject and stimulating the motor control learning process (Basteris et al., 2014; Proietti et al., 2016). When describing the cooperation between robot and human, in this study, we propose a terminology that describes the expected subject's behavior during interaction. For example, “Passive mode” will refer to subject-passive/robot-active training.

On top of these general definitions, it can be observed that one of the most critical areas in rehabilitation robotics is implementing the desired high-level modalities within the robot's hardware.

### 2.2. The Role of Compliant Control in Neurorehabilitation

Our concept relies on the concept of compliant and cooperative motion, i.e., the robot should behave transparently with respect to human activity, and eventually enhance user-driven movements. Compliant motion, by definition refers to the capability of the robotic system to generate movement and, simultaneously, undergo movement if external forces are applied. Typically, the perceived compliance can be implemented either through mechanical compliance, for example by using soft joints instead of rigid joints, or through compliant controllers (Calanca et al., 2016; Keemink et al., 2018). Moreover, these approaches intrinsically improve back-drivability and safety during human-robot interaction (Vallery et al., 2008).

From a low-level point of view, achieving compliant motion is a fundamental, yet challenging, task in rehabilitation robotics. In fact, if achieving the rigid behavior of the robot can be considered a trivial task, obtaining its opposite can be challenging since the low-level controller should reject the disturbances introduced by the robot hardware. At the same time, one of the key characteristics of the motor recovery process is not to limit, in any way, any intention of movement coming from the user and, possibly, of guiding the subject's voluntary movements toward the correct task execution. Compliant motion in rehabilitation robotics can, thus, be addressed as a compromise between good trajectory tracking and minimization of interaction forces.

Usually, rehabilitation robots and exoskeletons are provided with high-ratio transmission gearboxes that are kinematically inefficient, and that can introduce static and viscous friction. In this scenario, the perceived compliance cannot be guaranteed by the back-drivability of the motor itself. Still, it can be implemented by adding an elastic element in series with the actuation unit, i.e., series elastic actuators (SEA) (Crea et al., 2016; Calanca et al., 2017; Chen et al., 2019; Wu et al., 2019) or with compliant controllers that add virtual springs and dampers to shape the virtual mechanical impedance at the joint.

In the literature, several low-level controllers have been proposed to achieve compliant motion, and in turn, to implement the previously described training modalities. Among all, impedance control is one of the most common approaches, and it has been demonstrated to be a very efficient solution for neurorehabilitation (Mehdi and Boubaker, 2012). The impedance control belongs to those control schemes that permit a compliant pHRI. It implements dynamic control that relates force/torque and position: a torque/force output is generated from a position input. In particular, impedance control is characterized by a nested loop architecture. An inner torque-feedback loop implements the transparent behavior and promotes mechanical compliance (i.e., it “softens” the control). An outer position-feedback loop corrects for trajectory tracking errors by applying forces or torques aimed at the completion of the task (i.e., it “stiffens” the control). Furthermore, two different variants of the impedance control can be identified. When the actuation unit is inherently back-drivable, the torque control can be implemented through an open-loop current control loop (i.e., implicit impedance). In the other cases, a load-cell or an elastic element is exploited in series as a feedback signal for the closed-loop torque control loop (i.e., explicit impedance) (Calanca et al., 2016; Schumacher et al., 2019).

### 2.3. Available Control Strategies for Upper-Limb Exoskeletons

Regarding the rehabilitation domain, both impedance controllers in joint-space (Pehlivan et al., 2015; Just et al., 2017; Kim et al., 2017) and the Cartesian-space have been developed (Krebs et al., 2003; Frisoli et al., 2009; Ruiz et al., 2009; Mao and Agrawal, 2012). In joint-space impedance, the virtual mechanical elements are implemented in the joint-space with torsional spring and damper. The compliant behavior is given independently at each joint of the robot. Instead, with the Cartesian-space controller, virtual linear springs and dampers are connected to the robot end-effector in three-dimensional directions. Each direction is responsible for one of the three dimensions of the impedance ellipsoid computed at the robot end-effector. For example, in Kim et al. (2017), the baseline low-level control strategy of the Harmony robot, which is a bimanual upper-body exoskeleton for post-stroke rehabilitation, is based on a SEA-based joint-space impedance control that promotes the coordinated motion of the shoulder, through the assistance of the scapulohumeral rhythm (Kim and Deshpande, 2015). Specifically, for each joint, the deformation of the elastic element is used to estimate the generated torque at the joint axis. Then, an outer position-feedback is added to correct for task deviation. The dynamic model of the exoskeleton is formulated with a recursive Euler-Newton algorithm, and a feedforward term is added to compensate for gravity, friction, and dynamic torques. Similarly, the ARMin exoskeleton (Nef et al., 2007; Guidali et al., 2011; Just et al., 2017) is another example of an upper-limb exoskeleton for post-stroke rehabilitation based on a Proportional-Derivative (PD) position-feedback control that supports both the arm weight and provides assistance to the movement by virtually constraining the motion through stiffness/damping guidance. On top of this controller, the authors included online adaptive compensation algorithms to compensate for friction, elastic elements, and gravity terms (Just et al., 2018, 2020). On the other side, Frisoli et al. (2009) developed a Cartesian-space impedance-controlled exoskeleton to discriminate the end-effector reference trajectory from its orthogonal trajectory. In detail, two concurrent low-level impedance controllers act along the tangential and orthogonal directions of the trajectory, providing different virtual stiffness levels along with such directions and promoting a virtual tunnel that follows the Cartesian-space desired trajectory. Further evolution of impedance-based controllers involves the adaptation of the assistance according to the performances of the subject (Pehlivan et al., 2015; Perez-Ibarra et al., 2015; Pérez-Ibarra et al., 2019). Proietti et al. (2015) developed an exoskeleton controller based on adaptive techniques that can actively modulate the stiffness of the robotic device in function of the subject's activity. Instead, Pneu-WREX researchers developed a model-based adaptive control that learns from the patient's ability and provides support in completing movement while guaranteeing mechanical compliance (Wolbrecht et al., 2008). They implemented a Cartesian-space impedance control law, to which they added a feedforward term characterized by a non-linear sliding mode control scheme. The assistance-as-needed adaptation was achieved by adding a learning factor, which iteratively corrects the feedforward contribution, and a force decay, which reduces the support when the subject is able to perform the movement correctly.

This study identifies a compliant control framework that implements multiple high-level human-robot interaction modalities with a unique low-level explicit impedance control law. A similar compliant controller has already been implemented in other robots for neurorehabilitation (de Oliveira et al., 2019). Researchers already proposed that a mixture of assistance, correction, and resistance with impedance control laws could be used to gradually increase the amount of expected voluntary muscle activity. However, the generalization and validation of these approaches through the assessment of human volitional activity is still lacking.

To this aim, we employ an impedance-based controller to render different human-robot interaction modalities, and we demonstrate that the proposed controller can induce different levels of subject participation. We validate this approach by measuring the muscular voluntary effort of healthy volunteers through surface electromyographic (sEMG) monitoring. The experiments consist of elbow flexion/extension tasks executed under six different assistance and resistance levels by 14 healthy participants, who are instructed to self-tune their volitional contribution according to the effort needed to fulfill the tracking task.

## 3. Unified Compliant Control Framework

In this section, we introduce a compliant control framework based on an explicit impedance control law, capable of fulfilling different requirements and features, such as favoring good transparency of the joint, compensating for the weight of the robot and the supported limb, assisting the motion along the desired trajectory, recovering from task deviations, or even challenging the user by applying resistance or increased gravity to the motion.

**Figure 1:**
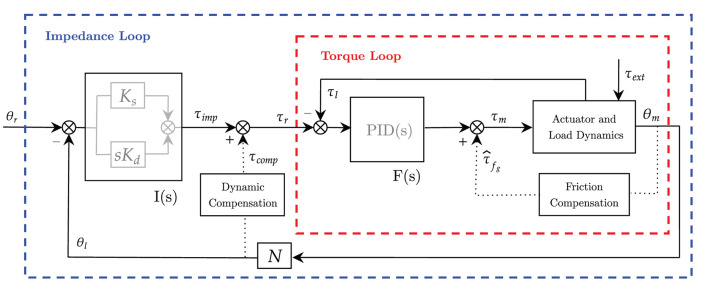
Block diagram of the unified controller scheme based on explicit impedance control law. Inner torque control *F*(*s*) is in red and outer impedance control *I*(*s*) is in blue. Dotted lines represent positive-feedback compensations.

The controller relies on the concepts of compliant control and, in particular, impedance control. The overall scheme of the proposed controller is presented in Figure 1. The idea is to employ a control architecture based on multiple nested control loops.

The outer impedance loop implements the virtual mechanical impedance *I*(*s*), which is in charge of correcting for deviations from the desired angular position and providing the anti-gravity compensation of the robot-human system. Namely, the outer loop provides the force-field assistance toward the completion of the task. We expect this assistance to be adaptable according to the desired training mode.

The inner torque loop *F*(*s*) is in charge of controlling the torque output at the load axis. It is aimed at promoting compliant behavior (i.e., mainly rejecting friction) and guaranteeing high-fidelity torque control. The inner torque control loop is employed to obtain an “explicit” feedback signal of the torque generated by the motor that rejects friction disturbances.

While the inner torque loop is supposed to be fast enough to neglect its dynamics, and its control parameters are kept fixed to exhibit stability, the outer loops (e.g., impedance loop and gravity compensation term) are characterized by adjustable parameters, to be adapted according to the desired pHRI.

**Figure 2:**
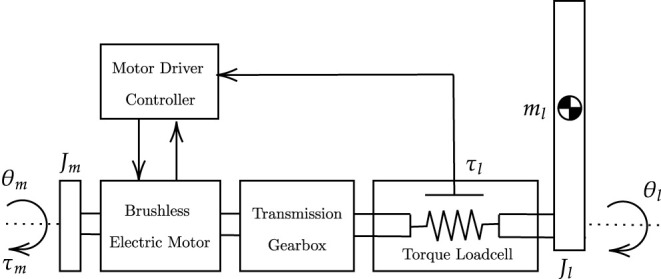
Actuation chain model. The actuation chain consists of an electric motor provided with an angular encoder, a transmission gearbox, a torsional torque sensor and an aluminum bar load. The motor driver acquires input signals from the actuation chain and commands torque set-points to the electric motor.

In this study, we consider an exemplary single-degree-of-freedom joint, shown in Figure 2, as a platform to validate the controller and its functionalities as it interacts with the human arm. The actuation chain is composed of an electric motor coupled with a high-ratio transmission gearbox. The unit is also provided with an incremental encoder that measures the joint angle, and a reaction torsional load-cell provides torque feedback at the output load axis. The dynamics of the one degree-of-freedom actuation system is as follows:


(1) $$\begin{array}{l} \tau_{l}=\left(\tau_{m}-J_{m}{\ddot{\theta }}_{m}-\eta_{m}{\dot{\theta }}_{m}-\tau_{fg}\right)N+\tau_{ext} \end{array}$$

where θ_*m*_ is the motor displacement, τ_*m*_ and τ_*l*_, respectively, represent the motor torque and the load torque measured at the load-cell, and τ_*ext*_ is the externally applied torque. The generated motor torque τ_*m*_ is converted in the acceleration of the rotor (${\ddot{\theta }}_{m}$) with inertia *J*_*m*_ in the dissipation of the motor damping η_*m*_ and friction τ_*fg*_ of the transmission gearbox. The resulting torque is then amplified by the gear ratio *N* and transferred to the output axis (θ_*l*_ of Figure 2).

### 3.1. Torque Control (Inner Loop)

The inner torque loop of an impedance controller can be implemented both as an open-loop (i.e., implicit impedance) or a closed-loop (i.e., explicit impedance) torque controller. In literature, Hogan first presented an implicit impedance controller that exploits an open-loop torque controller based on motor current control (Hogan, 1985, 1989). However, it requires inherent back-drivability, that can only be achieved with the low-ratio transmission or direct-drive actuators (Calanca et al., 2016). Several other approaches are available to compensate for undesired gearbox inefficiency. Model-based force estimation (Wolbrecht et al., 2008) or disturbance observer-based control schemes (Just et al., 2018) are common solutions. More often, torque sensors can be used to explicitly measure the actual generated torque and/or the subject's applied effort to be used as feedback in a closed-loop formulation (Focchi et al., 2016; Masud et al., 2018). In our study, since we consider high-ratio transmissions and the open-loop formulation would require a good friction model to achieve high-fidelity torque control, we opted for torque-controlled joints that are provided with torsional load-cells at each joint. In fact, torque-controlled robots are capable of producing very low impedance, which is essential to encourage users' voluntary contribution. In this form, the inner torque control *F*(*s*) is in charge of making sure that the measured torque output (τ_*l*_) follows the outer loop control variable (τ_*r*_). From the reference torque level to be actuated (τ_*r*_), the inner torque loop estimates the target torque of the actuator (τ_*m*_) through a Proportional-Integrative-Derivative (PID) controller, with feedback from the torsional load-cell (τ_*l*_), that in the Laplace form is (2):


(2) $$\begin{array}{l} F\left(s\right)=K_{p}+K_{i}/s+K_{d}s \end{array}$$

To compensate for static and viscous friction introduced by high-ratio gearboxes, an additional feedforward friction compensation (${\hat{\tau }}_{f_{g}}$), modeled as in Wit et al. (1998), has been added at the inner loop level, as shown in Figure 1. The compensation can be divided into Coulomb friction and velocity-dependent friction:


(3) $$\begin{array}{l} {\hat{\tau }}_{f_{g}}=\tau_{c}\tanh\left(\dot{\theta }/\dot{\theta_{c}}\right)+f_{v}\dot{\theta } \end{array}$$

where τ_*c*_ is the Coulomb friction torque, $\dot{\theta }$ is the measured joint velocity, $\dot{\theta_{c}}$ is the Coulomb joint velocity threshold, and *f*_*v*_ is the viscous friction coefficient. The hyperbolic tangent function ensures the Coulomb term to be continuous and smooth across $\dot{\theta }=0$ in order to avoid undesired oscillations. The $\hat{\tau_{f_{g}}}$ term is summed up to the torque PID control signal and fed as input to the actuator current control. The actual torque actuated at the load axis is then measured by the load-cell (τ_*l*_) and fed back to the PID controller to track the reference torque (τ_*r*_).

According to Calanca et al. (2016), the inner torque loop dynamics should not influence the outer loop. Thus, the inner loop is usually implemented at a higher control frequency. In our study, as previously stated, the torque loop is supposed to be fast enough to neglect its dynamics with respect to the outer impedance loop. Consequently, the torque control loop should be considered an ideal torque source and only serves as a baseline for the impedance control loop.

As suggested by Focchi et al. (2016), the parameters tuning of the explicit inner torque loop strongly influences the stability of the system. We decided to empirically tailor the inner controller to exhibit stable behavior throughout the full range of achievable impedance at the outer loop. For this reason and to avoid unstable conditions, the inner loop is operated with fixed parameters, which are considered constant regardless of the desired high-level mode.

### 3.2. Impedance Control (Outer Loop)

The impedance control can be regarded as an outer position loop that takes a reference in terms of angular position (i.e., θ_*r*_) and, by means of a virtual mechanical impedance, produces a reference torque (i.e., τ_*r*_) that in turn is fed to the inner torque loop. The total reference torque can be seen as composed of two contributions, as in Equation (4). The feedback impedance-based term, namely τ_*imp*_, corrects for tracking errors and dampens undesired oscillations. The feedforward term τ_*comp*_ compensates for the dynamic model of the robot and the weight of the wearer's limb.


(4) $$\begin{array}{l} \tau_{r}=\tau_{imp}+\tau_{comp} \end{array}$$

Instead, the measured torque at the load axis consists of the actual torque generated by the robotic system and can be broken down into four main components, as shown in Equation (5):


(5) $$\begin{array}{l} \tau_{l}=\tau_{comp}+\tau_{imp}+\tau_{ext}+\tau_{res} \end{array}$$

where τ_*comp*_ and τ_*imp*_ represent the actuation torques commanded to the motor, τ_*ext*_ indicates the external torque that the user exerts to the joint, and τ_*r*_*es* represents the residual disturbance torque that the inner torque controller can not reject.

#### 3.2.1. Feedback Impedance-Based Term

To derive the feedback impedance-based term (i.e., τ_*imp*_), considering a first order impedance, the transfer function I(s) between the reference target (θ_*s*_) and the impedance-based torque term (τ_*imp*_) is characterized by two parameters: virtual spring (*K*_*s*_) and virtual damper (*D*_*d*_), and it can be implemented in the well-known form Equation (6):


(6) $$\begin{array}{l} I\left(s\right)=K_{s}+sD_{d} \end{array}$$

that in the time domain becomes Equation (7):


(7) $$\begin{array}{l} \tau_{imp}=K_{s}\left(\theta_{d}-\theta \right)+D_{d}\left({\dot{\theta }}_{d}-\dot{\theta }\right) \end{array}$$

where τ_*imp*_ is the desired impedance control torque that is used as a set-point by the inner torque loop, while θ_*d*_ and θ are, respectively, the desired and measured joint angle positions.

**Figure 3:**
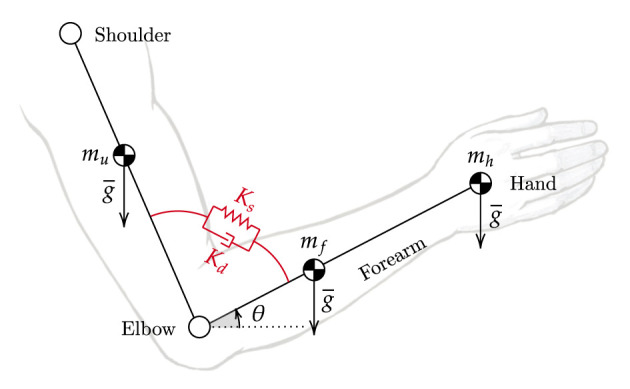
Impedance model at the elbow joint. The unified controller implements virtual stiffness and damping (in red) at the elbow joint. The elbow joint is rendered as a second-order system mass-spring-damper. The equilibrium point of the spring continuously changes according to the desired trajectory (θ).

The virtual stiffness, by means of the virtual spring constant *K*_*s*_, pulls the joint link toward its desired configuration (i.e., the spring corrects for deviations from its equilibrium point, which is continuously adapted to follow the desired angular trajectory). At the same time, the virtual damper (*D*_*d*_) dissipates the spring energy and damps oscillations. Overall, the role of these parameters is to render, as shown in Figure 3 for the elbow joint, a second-order system by virtualizing a spring-damper component within the impedance control law. When dealing with robotic rehabilitation, the desired angular velocity might not be available, especially when the task trajectory is updated in real-time to follow the subject's intention of movement. In such cases, we can neglect the reference velocity term (${\dot{\theta }}_{d}$) in the previous (Equation 7). In this way, the damping term is related to the absolute velocity instead of the error velocity. The virtual damper is fixed to the ground frame, resulting in always-resistive damping of the system.

#### 3.2.2. Feedforward Weight and Dynamics Compensation Term

A feedforward torque reference term that accounts for the dynamics of the robot and for weight of the arm is added at the impedance control level.

For the sake of simplicity, in this section, we consider the general single-degree-of-freedom joint shown in Figure 3, which can be reduced to a rigid pendulum system. The torque acting at the load axis can be described with the dynamic equation of the system, which includes both the robot and the human, as follows Equation (8):


(8) $$\begin{array}{l} \tau =J_{l}\ddot{\theta }+f_{l}\dot{\theta }+G\left(\theta \right) \end{array}$$

where *J*_*l*_ is the inertia moment, *f*_*l*_ is the viscous friction at the load axis, and *G* represents gravitational torques for both the link and the forearm. Compensating for the inertial component of the dynamic model requires the estimation of inertia moments and the computation of the acceleration by twice-differentiating the encoder position. These operations can raise many difficulties and undesired uncertainties that are in turn fed to the controller as positive-feedback terms. Inertia compensation can, thus, make the system non-passive and can jeopardize the coupled stability of the human-exoskeleton system (Kim et al., 2014). Additionally, in robotic rehabilitation, the desired arm movements are usually slow, leading to neglectable effects due to the dynamic terms of Equation (8). For these reasons, in our study, we only compensate for gravitational and viscous frictional torques.

We, therefore, introduce the simplified compensation term:


(9) $$\begin{array}{l} \tau_{comp}=\hat{f_{l}}\dot{\theta }+{Ĝ}_{link}\left(\theta \right)+{Ĝ}_{wc}\left(\theta \right) \end{array}$$

where $\hat{f_{l}}$ is the estimated viscous friction coefficient, Ĝ_*link*_ represents the weight compensation term for the robot components, and $\hat{G_{w}c}$ represents the weight compensation of the human component. The weight compensation term for the robot can be modeled as in Equation (10):


(10) $$\begin{array}{l} {Ĝ}_{link}\left(\theta \right)=mgl\cos\theta \end{array}$$

where *m* is the robot link mass, *l* its center-of-mass distance, and *g* is the gravitational acceleration.

As for the gravitational compensation term of the human (Ĝ_*wc*_), we need to make explicit reference to the single-degree-of-freedom joint used as a demonstrative example (Figure 3). Of course, this can be generalized to any joint of interest. We have included vertical forces applied at the centers of mass of the forearm and hand. The position of the center of mass and the weight of the limbs can be derived from the anthropometric tables presented in Winter (2009). The level of weight assistance can be regulated by means of a weighting factor (ranging from 0 to 100%) that accounts for misalignment and uncertainties in the anthropometric data as in Equation (11):


(11) $$\begin{array}{l} {Ĝ}_{wc}\left(\theta \right)=W_{f}\left(m_{f}l_{f}+m_{h}l_{h}\right)g\cos\theta \end{array}$$

where *W*_*f*_ is the weighting factor, *m*_*f*_ and *m*_*h*_ are the masses of forearm and hand, while *l*_*f*_ and *l*_*h*_ are their centers of mass. With this dynamic compensation, only inertial, centrifugal, and residual frictional torques are to be overcome if the user wants to perform a voluntary movement (i.e., they are not included in the compensation term).

The feedforward compensation torque formulation can be obviously generalized if an *n*-degree-of-freedom robot is concerned. In such cases, the dynamics compensation terms can also include Coriolis and centrifugal torques. Such feedforward compensation can be computed from centralized inverse dynamics algorithms, such as closed-form solutions or more computationally efficient recursive Euler-Newton approaches (Moubarak et al., 2010; Ragonesi et al., 2013; Kim et al., 2017; Just et al., 2020).

### 3.3. Human-Robot Interaction Modalities

In this study, we took inspiration from literature control modalities for robot-mediated therapy, and we selected six high-level human-robot interaction modalities, ranging from passive mobilization to challenging modalities. In this section, we first describe the motor learning rationale and the desired high-level behavior for each mode. Then, we propose a match between the high-level behavior and a set of control parameters that can render the desired behavior. The claim regards the adjustment of stiffness, damping, and weight-compensation assistance to render different pHRI levels. We underline that the parameters are adapted only at the higher level as the torque control loop serves as an internal loop to promote compliant behavior and improve the torque tracking accuracy.

#### Passive Mode (P)

The *P* mode should be exploited during the preliminary stages of the rehabilitation process. The robot helps the patient to track a predefined trajectory to improve the limb range of motion and reduce muscular atrophy or tendon retractions (Masiero et al., 2007). When the system is operated in *P* mode, the robot performs the movement without accounting for the user's intentional activity. Stiffness *K*_*s*_ and damping *D*_*d*_ control gains are greater than in other modes, rendering a stiffer behavior of the joint, and the torque feedforward term (τ_*comp*_) is used to compensate for the user's arm weight. However, in this mode, the trajectory tracking is not as accurate as in position control, as the impedance control intrinsically introduces a tolerance dead-band. Nevertheless, this is not a critical aspect for rehabilitation robots since the crucial feature is to limit the interaction forces with the human limb.

#### Corrective Mode (C)

*Corrective* modalities are used when patients have some voluntary muscular contractions, but the generated strength is not sufficient to perform complete and functional movements. The robot provides assistance when the participant is not capable of fulfilling the task, and generates a force-field to push the arm toward the desired path (Basteris et al., 2014). In this mode, subjects generate the minimum effort needed to accomplish the desired task. The user is actively involved in the movement, and the robot partially assists the motion. The *C* mode is implemented with impedance-based assistance. Lower values for both virtual stiffness and damping are used with respect to the *P*, rendering a more compliant and softer behavior of the robot, i.e., the user is allowed to deviate from the trajectory.

#### Weight Counterbalance Mode (W)

The *W* mode can be applied to perceive a microgravity environment. This effect is obtained through the counterbalancing assistance term that is computed according to the configuration of the user's arm. In this mode, the subject is wholly involved in the task, and if the voluntary activity is not sufficient to fulfill the exercise, the robot does not apply for any corrective assistance. Indeed, the controller is not programmed to follow a predefined exercise task. At low-level, the virtual stiffness is removed, and a low damping value is used to avoid undesired oscillations and dampen the motion.

#### Transparent Mode (T)

In *T* mode, the user performs the task, and the robot follows the movement without assisting (nor resisting) the movement. In other words, this mode enables the robot to be dynamically transparent to users' voluntary movements, by compensating the exoskeleton weight at each configuration along with the task. Regarding its implementation, the low impedance behavior is achieved by means of a null-torque controller provided only with the compensation for the robot weight. Neither assistance nor resistance is provided.

#### Resistive Mode (R)

The *R* mode has been introduced to further engage the patient along with his/her progression, i.e., when most of the motor functionalities have been (hopefully) relearned, but the subject still has to gain some muscular tone. In fact, robots with torque-controlled joints can also realize an aquatic therapy-like environment by providing weight support and allowing user-driven free motions with or without viscous resistance (Kong et al., 2010; Song et al., 2014). To implement such behavior, this mode adds a viscous-like resistance to the movement while compensating for the robot dynamics. No impedance-based assistance is present, and the controller resists the movement by applying a viscous frictional torque, which is inversely proportional to the movement velocity.

#### Hypergravity Mode (H)

The *H* mode amplifies the effect of gravity during the movement. This mode can be used to challenge the subject during the exercise and to focus the training on postural anti-gravity muscles. In particular, instead of counterbalancing the limb weight, the controller adds additional virtual weight, applied at the centers of mass of the limb, that gives the feeling of doing the task with weight, or, in other words, of doing the exercise in a hyper-gravity environment.

Overall, qualitative guidelines suggest that high-impedance implementation should be used to stiffen the control law, imposing the subject's movement along the task trajectory. Contrarily, low-impedance gains should be exploited to render more compliant and softer behavior of the robot, i.e., the controller promotes voluntary movements, and the user is allowed to deviate from the trajectory. Finally, we usually increased the damping not only to reduce overshoots and oscillations but also to introduce a baseline kinematic error, which should engage the user when following the desired trajectory. However, a trade-off in the impedance parameters is needed to induce a physiological muscular activation aimed at completing the task in an assisted-as-needed fashion. Regarding the weight counterbalance, the mathematical model does not always entail a real experience of weight relief for the end-user. As a consequence, we adapted the level of anti-gravity support by means of the weighting factor *W*_*f*_, which was tuned to 75%. Indeed, in the implementation of anti-gravity exoskeletons, a 100% compensation could hinder the user during anti-gravity movements, and it is suggested to compensate for a fraction of the full dynamics (Näf et al., 2018).

To define the quantitative values of stiffness, damping and weight assistance for each mode, we separately ran some preliminary tests on two healthy subjects, which were not recruited for the rehabilitation modalities assessment to avoid bias. The parameters of the controller were empirically determined according to the perceived behavior.

Table 1 shows the parameters that we used for the human-robot interaction modalities validation, as described in section 3.3.

**Table 1:** The proposed parameters used with the unified compliant controller to render the selected high-level human-robot interaction modalities.

**Human-robot interaction modalities**		**Weight (*W*_*f*_)**	**Stiffness (*K*_*s*_)**	**Damping (*D*_*d*_)**
		**(%)**	**(Nm/rad)**	**(Nms/rad)**
Passive	P	75	50.0	10.0
Corrective	C	0	5.0	1.0
Weight counterbalance	W	75	0.0	0.1
Transparent	T	0	0.0	0.1
Resistive	R	0	0.0	3.5
Hypergravity	H	–100	0.0	0.5

*K_s_ and D_d_ relate to the impedance-based term. The W_f_ parameter corresponds to the weighting factor for the feedforward compensation of Equation (12) and it was manually tuned to 75% to avoid overcompensating for the user's arm weight*.

## 4. Methods

To assess the validity of the proposed control framework and its ability to promote different human-robot interaction modalities, we considered a typical actuation joint for a general upper-limb exoskeleton, and we used it as a platform to test and verify the performances of the proposed controller. As previously explained, the controller is first characterized regardless of the volitional human activity, then the perceived pHRI is assessed on elbow flexion/extension movements throughout the proposed modalities. In detail, the validation of the control framework is presented in two different steps: i) assessment of the performances of the control loops 4.2, and ii) electromyographic validation of the unified compliant control operating in the proposed rehabilitation modalities 4.3.

### 4.1. Experimental Set-Up

**Figure 4:**
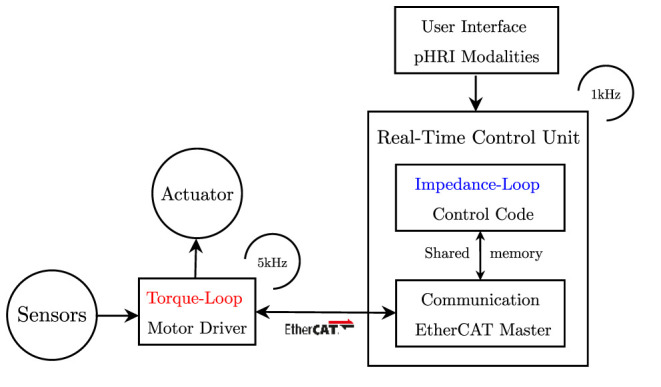
Control framework block diagram. The inner torque loop is implemented in the motor driver. The outer impedance loop is implemented in the real-time control unit. Configuration files are used to personalize the controller parameters. The user can select the rehabilitation mode through a simple user interface.

The actuation is provided by a brushless DC motor (EC-45 flat, Maxon Motor AG, Switzerland), coupled with a planetary gearhead with a transmission ratio of 156:1 (GP-42-C, Maxon Motor AG, Switzerland). The electric motor provides a nominal torque of about 120 mNm. Thus, given the ratio and the efficiency of the transmission, the gear motor is able to provide at the load side a maximum constant torque of about 15 Nm and a peak torque of about 18.5 Nm. An incremental encoder reads the rotor position with a resolution of 2,048 counts per revolution, leading to a resolution of 0.001° at the load side. Finally, a reaction torsional load-cell (TRT-200, Transducer Techniques, CA, USA) is connected to the gearbox output shaft to sense the torque acting on the joint of the robot. With the aim of testing the control in the interaction with the human motion, we designed a one-degree-of-freedom robotic system to provide assistance to the elbow during flexion-extension tasks, similar to the one presented by Lobo-Prat et al. (2016). In particular, the rotational axis of the system is aligned with the user's elbow joint. An aluminum bar is attached to the load-cell and is fixed to the user's forearm by means of an ergonomic arm cuff. The arm cuff is equipped with padded fabric which minimizes interaction forces between the rigid shell and the arm. Adhesive strips are used to fix it to the arm cuff. The user's elbow rests over a soft foam surface, and the arm cuff position can be adjusted according to the forearm length to improve the comfort and alignment of the rotation axis. The unified controller described in section 3 is implemented in a real-time control system, based on Linux patched with PREEMPT RT, and runs at a cycle time of 1 ms. The control hardware architecture shown in Figure 4 relies on the EtherCAT field-bus, which guarantees good performances on distributed networks, and assures a reliable, deterministic, stable, and low-latency communication between the control unit and the connected hardware. In particular, the motor driver (Mini Torque Driver, Esmacat, US) is connected to the control system *via* the EtherCAT communication, and a real-time C++ master application, based on the Simple Open-Source EtherCAT Master (SOEM) library, is implemented to handle the communication with the motor and sensors. The real-time control unit also implements the outer impedance/position loop at 1 kHz, the feedforward compensations, and the trajectory generation. The low-level torque control is instead implemented in the motor driver at 5 kHz.

**Figure 5:**
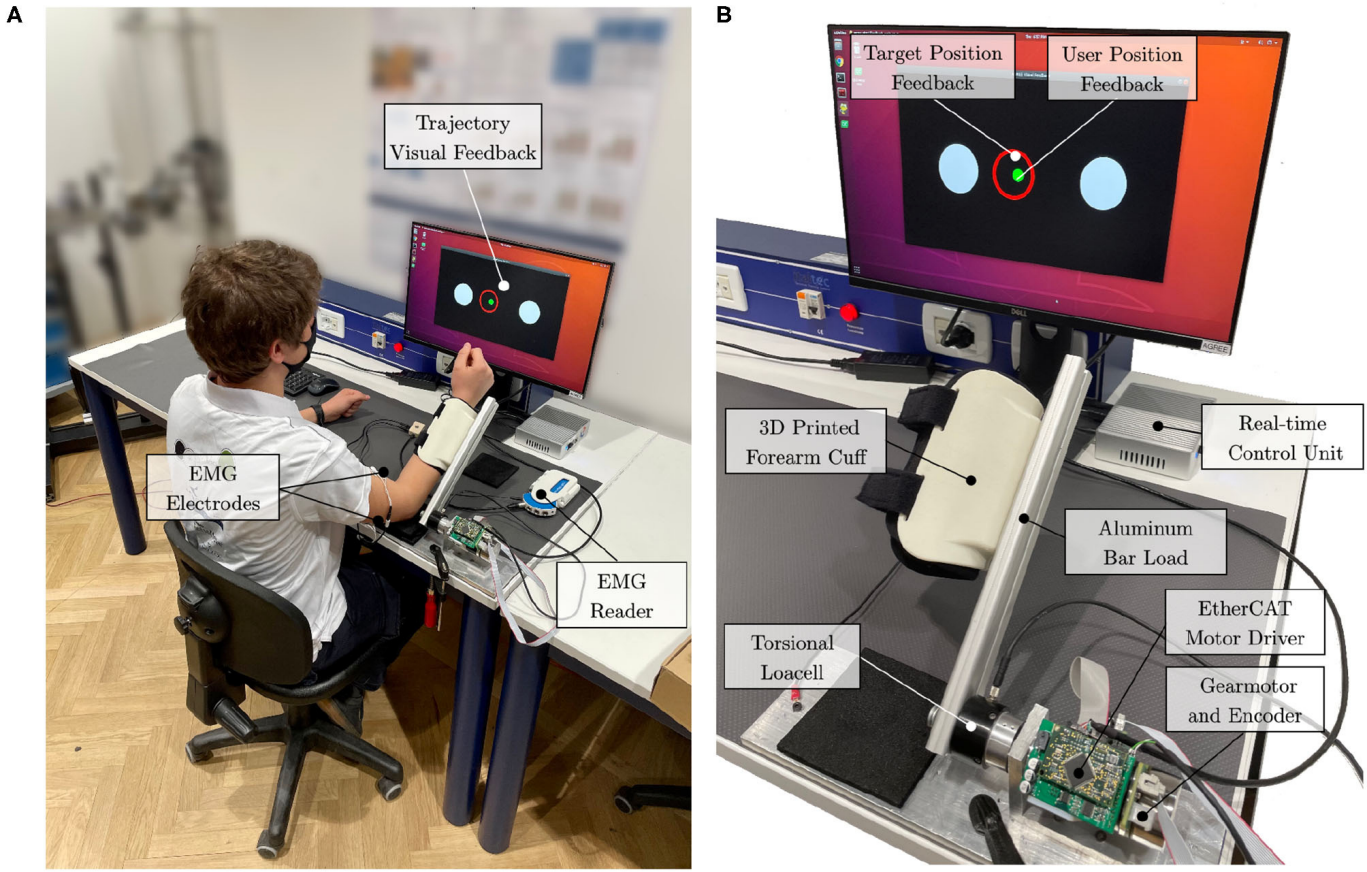
Experimental setup. **(A)** The subject is attached to the elbow-joint system at the forearm. The elbow leans on the table over a soft foam surface. Surface electrodes are placed at the *biceps* and *triceps brachii* (long head). **(B)** The actuation drive system is connected to the real-time control unit with EtherCAT. Visual feedback is provided to the user to help follow the desired trajectory.

The experimental set-up and its connection are described in Figure 5B, while its final realization is shown in Figure 5A. The main features of the presented experimental set-up are reported in Table 2.

**Table 2:** The main features and specifications of the developed experimental set-up.

**Specifications**	**Value**
Nominal torque	15 Nm
Max. Peak torque	18.5 Nm
Max. Velocity	4.4 rad/s
Gearbox ratio (*N*)	156:1
Range-of-motion	0° +110°
Torque control frequency	5 kHz
Impedance control frequency	1 kHz

*Performances of the torque control and impedance control loops are discussed in section 5.1*.

### 4.2. Actuation and Control Characterization

The characterization of the controller was conducted on a single healthy subject as its performances are to be assessed regardless of the subject's performance and involvement.

First, we assessed the capability of the system to promote physical human-robot transparency, defined in literature as to how good the robot is at rejecting torque disturbances and at limiting resistance during subjects' voluntary motion (Zanotto et al., 2013). To validate the need of employing the inner torque closed-loop and consequently to assess the ability of the unified compliant controller to improve transparency, open-loop (current-based) null-torque control was compared to closed-loop (loadcell-based) null-torque control. To this aim, we asked a healthy subject to perform movements spanning the whole available range of motion (i.e., 0° to +90°) with the elbow one-degree-of-freedom test-bed at various velocities ranging from –1.0 to 1.0 rad/s in two conditions: i) back-driving movements operating the joint with no active control. In this condition, the inner loop is disabled and the mechanical backdrivability of the joint is sensed; ii) back-driving movements operating the joint in closed-loop null-torque control: The inner torque control follows a null torque reference. We measured the torque output from the torque sensor, while the position and velocity of the joint axis were obtained from the embedded incremental encoder. We computed the maximum residual resistive torques, which should be lower for better transparency.

Second, to assess the accuracy of the torque control, we analyzed the frequency response of the inner closed-loop. We set the drive system at its mechanical end-stop, and we commanded sinusoidal torque profiles to the actuator torque control at different frequencies, ranging from 0.5 to 4 Hz. We evaluated the differences between the commanded torque and the measured torque curves using Root Mean Squared Error (RMSE) and Peak Error (PE) values, which are measures of the accuracy of the torque-control loop and both should be as small as possible. Finally, we computed the Pearson correlation coefficients to evaluate torque fidelity at each frequency, which should be greater than 90% for high similarity levels (Mukaka, 2012). At last, we investigated the performance of the impedance control, and we estimated the accuracy of the rendered torsional impedance values that the system was able to generate. The robot was commanded in impedance at the vertical equilibrium point (θ = 0°), and external torques were exerted to the joint-link system. The experiment was repeated at different stiffness/damping values. The displacement from the equilibrium point (in radians) at stiffness values of 5, 10, 20, and 40 Nm/rad has been evaluated and related to the measured torque output. One should verify that the experimental stiffness matches the commanded one.

### 4.3. Human-Robot Interaction Modalities Validation

The testing protocol was performed on healthy subjects, and it was approved by the ethical committee of Politecnico di Milano.

The protocol involved the execution of elbow flexion/extension tasks with the elbow-joint developed set-up (Section 4.1). The system was connected to the dominant arm of the user, and the user performed elbow flexion and extension movements following the six implemented rehabilitation strategies. Their sequence was randomized to avoid learning or fatigue effects, that could have biased the results. For each mode, the user performed 15 elbow flexion/extension repetitions. The user was instructed to perform the movements following visual feedback (Figure 5A). The visual interface showed the movement to be tracked and the actual position of the joint. The desired movement speed was kept the same across all modalities.

The goal of the task was to correctly track a moving trajectory, and the performance was the resultant of the sum of the contribution of the torque provided by the human and the motor. In this view, healthy subjects can modulate their contribution to the movement. Thus, we have been able to monitor different human contribution levels while keeping the task performance constant.

**Figure 6:**
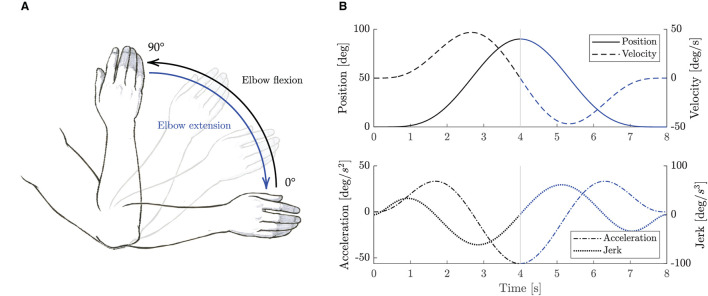
Elbow flexion/extension movements. **(A)** Sketch representation of the elbow flexion/extension task exercise. **(B)** Trajectory β-function computed with *P*0 = *P*2 = 0, *P*4 = 8, *A*0 = 90°, and *P*3 = *P*5 = 5. Black lines represent elbow flexion phase, while blue lines represent elbow extension phase.

As proposed in Hogan (1984), the movement of the human arm, when coupled with a robot, can be described by a minimum jerk trajectory. In this study, we defined the nominal trajectory by means of a symmetric fifth order β-function (Krebs et al., 1999) as in Equation 12. The nominal trajectory starts with the forearm lying on the table (i.e., 0°), then the flexion/extension movement is performed in about 8 s as in Figure 6A.


(12) $$\begin{array}{l} \theta_{r}\left(t\right)=P_{0}+P_{1}{\left(t-P_{2}\right)}^{P_{3}}{\left(P_{4}-t\right)}^{P_{5}},P_{2}\leq t\leq P_{4} \end{array}$$

(13) $$\begin{array}{l} P_{1}=\frac{A_{0}}{{\frac{P_{4}-P_{2}}{2}}^{\left(P_{3}+P_{5}\right)}} \end{array}$$

where the *P*_*n*_ parameters are used to configure the desired trajectory. *P*_0_ represents the initial position offset, *P*_2_ and *P*_4_ are the start and the stop time, *P*_3_ and *P*_5_ are the interpolators' orders for the raising and falling phases, and *P*_1_ is related to movement amplitude *A*_0_ by means of Equation (13). Figure 6B shows the desired β-function trajectory for the elbow flexion/extension movement.

#### 4.3.1. Outcome Measures

We recorded the kinematic and dynamic data from the robot sensors. Commanded and measured angular position, velocity and torques were sampled at a frequency of 1 kHz. Torque data were low-pass filtered with a Butterworth filter of the third order and a cut-off frequency of 20 Hz. To investigate how subjects adapted their motion control to various assistance (or resistance) levels, and to posit if the experiments were comparable, we assessed the kinematics variability. In particular, to evaluate if the subjects performed comparable trajectories across all modalities and, as a consequence, if we could posit that all the subjects performed the same movements, we computed the RMSE between the commanded and the measured angular position across all repetitions, subjects, and modalities.

To validate the implemented control strategies and to investigate how they affect the user's behavior, we also registered the muscular activity. In particular, we recorded the *biceps* and *triceps* (long head) muscles, as shown in Figure 5B. The sEMG signal was recorded at a frequency of 1 kHz with a wireless EMG reader (Sessantaquattro, OTbioelettronica, Italy). EMG signals were pre-processed following a standard approach that includes high-pass filtering with a third-order Butterworth filter at 10 Hz, rectification, and low-pass filtering with a third-order Butterworth filter at 4 Hz (Gandolla et al., 2018). We normalized signals for each participant with respect to 80% of the maximum contraction during the whole experimental session, preventing normalization by spurious EMG spikes (Ricamato and Hidler, 2005). We computed the integrated EMG (iEMG) as a marker of voluntary muscle drive as the area under the curve of the normalized EMG signal (Androwis et al., 2018).

#### 4.3.2. Statistical Analysis

Outcome measures were collected for each subject and for each control mode. All output indices were computed separately for the flexion and extension movements. Results are expressed as medians and inter quartile ranges (IQR) [25th - 75th percentiles]. Given the reduced sample size, the Friedman test was performed to detect possible significant changes in the RMSE and iEMG indices across different control strategies. *Post-hoc* comparisons with Bonferroni correction were used to identify statistically significant differences between the six modalities. All statistical analyses have been performed in MATLAB (version R2020b) and IBM SPSS Statistics (version 27).

## 5. Results

### 5.1. Actuation and Control Characterization Results

**Figure 7:**
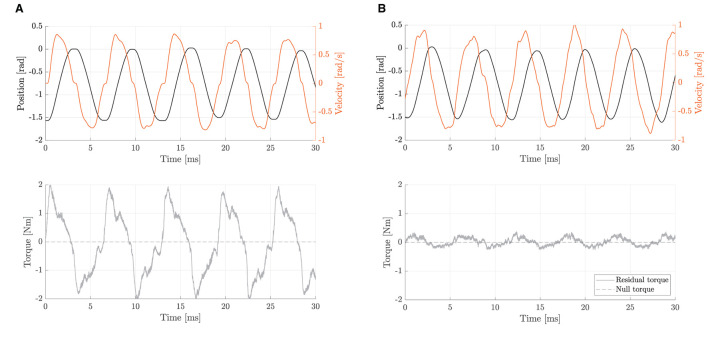
Backdriving movements during free-motion at velocities ranging from -1.0 to 1.0 rad/s. The residual torque represents the difference between commanded (null) and measured torques. **(A)** With the open-loop null-torque control, no active disturbance rejection is enabled. Residual torques are up to 2.0 Nm. **(B)** With the closed-loop null-torque control, the inner torque loop is enabled. Residual torques range from about -0.3 to 0.3 Nm.

As for the capability of the compliant controller to promote physical human-robot transparency, results demonstrated that the closed-loop torque controller reduced the residual frictional torques, from 2.0 to 0.3 Nm. As shown in Figures 7A,B, when the robot is operated in closed-loop null-torque control, better transparency is achieved within a range of –1.0–1.0 rad/s, which are typical maximum velocities for a rehabilitation exercise (Neilson, 1972). The maximum residual resistive torques during back-driving movements were perceived as negligible by the user that was performing the experiment. This result confirms that employing a loadcell-based torque control loop permits to achieve higher transparency of the joint and better torque tracking.

**Figure 8:**
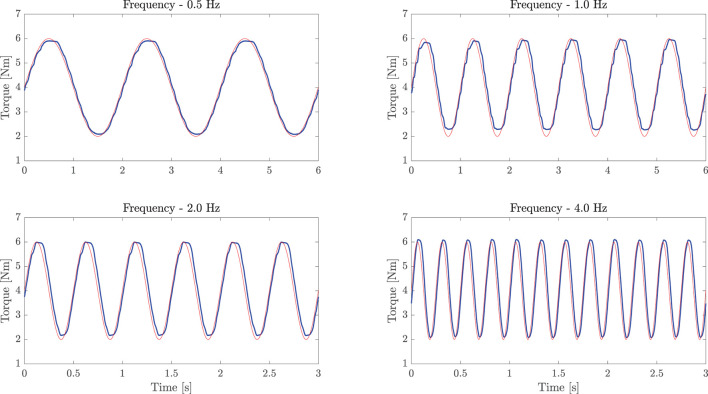
Sinusoidal torque response at different frequencies (0.5, 1.0, 2.0, and 4.0 Hz). The experiment demonstrates good torque tracking of the inner torque loop. The light red line represents the commanded torque, while the bold blue line refers to the load-cell measured torque.

To measure the torque control accuracy, we performed sinusoidal torque profiles, as shown in Figure 8. The differences between the commanded torque and the measured torque curves were computed to assess the accuracy of the inner closed-loop torque control. Results showed torque output RMSE of 0.12, 0.30, 0.33, and 0.49 Nm, respectively, for 0.5, 1.0, 2.0, and 4.0 Hz. The maximum (PE) of about 0.90 Nm was obtained at 4.0 Hz in correspondence to sudden changes (i.e., at the inversion of velocity). Pearson correlation coefficients resulted equal to 99.62 (f = 0.5 Hz), 98.06 (f = 1 Hz), 97.55 (f = 2 Hz), and 94.71% (f = 4 Hz), demonstrating a high-fidelity torque control.

**Figure 9:**
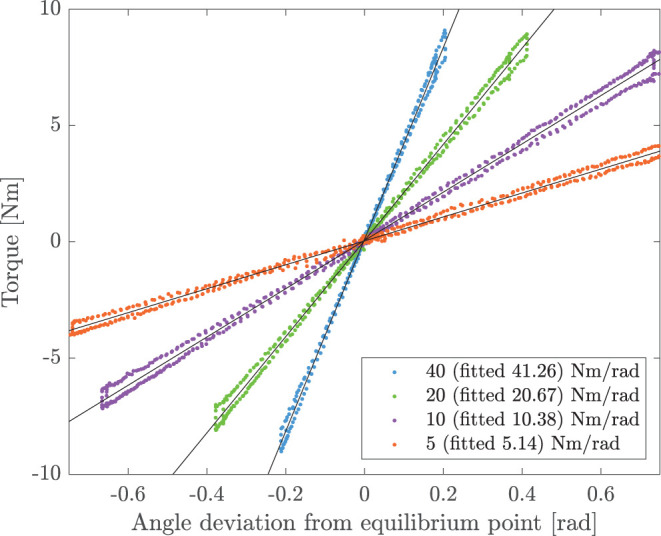
Experimental torsional stiffness. The joint is controlled in impedance at the equilibrium point (θ = 0°). The user exerts external forces to the joint. Torque and angle deviation are measured and compared at different virtual stiffnesses. For the commanded values of 5, 10, 20, and 40 Nm/rad, the measured experimental stiffnesses are 5.14, 10.38, 20.67, and 41.26 Nm/rad, respectively.

As for the performances of the impedance controller, Figure 9 shows the relationship between the generated torque output (in Nm) and the displacement from the equilibrium point (in rad) at stiffness values of 5, 10, 20, and 40 Nm/rad. Notably, the fitted values from the experimental data demonstrate a good stiffness accuracy, resulting in an average relative error of 3.3±0.3% with respect to desired values.

### 5.2. Human-Robot Interaction Modalities Validation Results

We assessed the capability of the controller to implement the proposed high-level modalities by measuring the perceived pHRI through the monitoring of the voluntary muscular effort of healthy participants. We recruited 14 healthy volunteers, with a median age of 25 years, [24-27] IQR.

#### 5.2.1. Kinematics Variability Assessment

The results of the trajectory tracking RMSE of the elbow joint reported that the overall average tracking error was 3.38 ± 1.29 degrees, and the maximum detected RMSE was 5.73 degrees (about 0.1 radians). The Friedman test rejected the null hypothesis that data came from the same distribution (*p* < 0.0001). The *post-hoc* analysis revealed that only RMSE data of the *P* mode significantly differed from all the other groups (*p* < 0.01). As expected, since we are using an impedance control logic, which does not guarantee an accurate position tracking, and since no effort was required from the user, in *P* mode, we can notice higher errors, but the trajectory variability is minimal. Finally, in *W* mode, by which the controller does not correct for trajectory deviation, the tracking RMSE was slightly higher than in the other modalities.

#### 5.2.2. Electromyographic Monitoring

**Figure 10:**
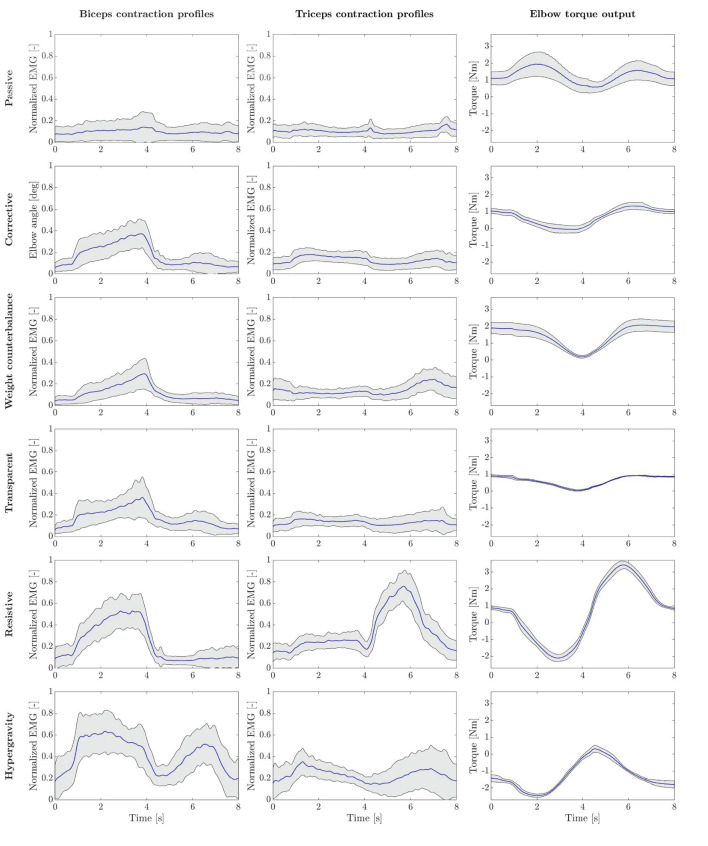
Experimental results for all the presented rehabilitation modalities. Each row represents a different mode. Subplots show *biceps* and *triceps* normalized EMG, and measured interaction torque, generated at the output joint axis. Bold blue lines represent mean values, while gray areas refer to SD ranges.

In Figure 10, we present the average envelope profiles of muscular contraction (*biceps* and *triceps brachii*), and the torque output for each of the presented high-level modalities.

**Figure 11:**
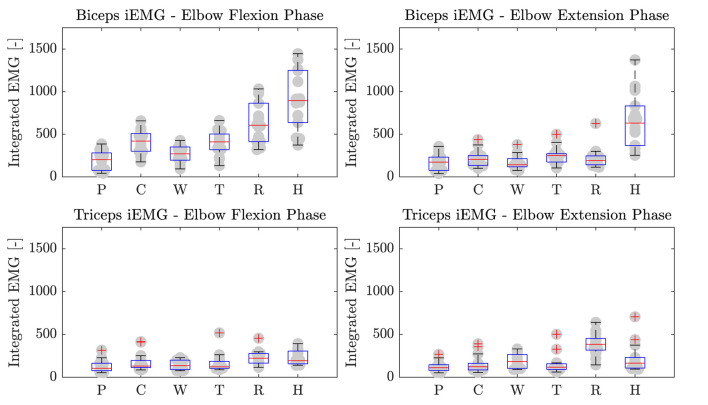
*Biceps* and *triceps brachii* iEMG during elbow flexion and extension phases. P, Passive mode; C, Corrective; W, Weight counterbalance; T, Transparent; R, Resistive; H, Hypergravity.

Furthermore, the iEMG results are reported in Figure 11 for each high-level mode. The Friedman test revealed significant differences among training modalities for the iEMG index for the four conditions analyzed (i.e., *biceps* and *triceps* contraction during elbow flexion and extension phases) (*p* < 0.0001). Therefore, we performed further analysis to separately compare each rehabilitation mode with the others. The results of the *post-hoc* analysis are shown in Tables 3, 4.

**Table 3:** *P*-values results of the *post-hoc* analysis comparing integrated EMG (iEMG) index among training modalities during elbow flexion movement.

**Muscular contraction during elbow flexion phase**						
**Biceps**	**P**	**C**	**W**	**T**	**R**	**H**
**P**	−					
**C**	**<0.001** ↑	−				
**W**	0.101	**<0.001** **↓**	−			
**T**	**<0.001** ↑	1	**0.002** ↑	−		
**R**	**<0.001** ↑	**<0.001** ↑	**<0.001** ↑	**0.001** ↑	−	
**H**	**<0.001** ↑	**<0.001** ↑	**<0.001** ↑	**<0.001** ↑	1	−
**Triceps**	**P**	**C**	**W**	**T**	**R**	**H**
**P**	−					
**C**	0.055	−				
**W**	0.620	0.831	−			
**T**	0.101	0.081	1.000	−		
**R**	**0.003** ↑	**0.014** ↑	**0.002** ↑	**0.002** ↑	−	
**H**	**0.005** ↑	**0.011** ↑	**<0.001** ↑	**0.001** ↑	1.000	−

*Bold values indicate significant differences between muscular contraction obtained with row and column modalities. Up and down arrows are used to specify if the mode represented in the selected row has significantly higher (↑) or lower (↓) muscular contraction than the mode represented in the respective column of the table*.

**Table 4:** *P*-values results of the *post-hoc* analysis comparing iEMG index among training modalities during elbow extension movement.

**Muscular contraction during elbow extension phase**						
**Biceps**	**P**	**C**	**W**	**T**	**R**	**H**
**P**	−					
**C**	1.000	−				
**W**	1.000	1.000	−			
**T**	**0.004** ↑	1.000	**0.013** ↑	−		
**R**	**0.043** ↑	1.000	0.125	1.000	−	
**H**	**<0.001** ↑	**<0.001** ↑	**<0.001** ↑	**<0.001** ↑	**<0.001** ↑	−
**Triceps**	**P**	**C**	**W**	**T**	**R**	**H**
**P**	−					
**C**	0.272	−				
**W**	0.101	1.000	−			
**T**	0.226	1.000	1.000	−		
**R**	**<0.001** ↑	**0.002** ↑	**<0.001** ↑	**0.006** ↑	−	
**H**	**0.013** ↑	**0.011** ↑	0.229	**0.004** ↑	**0.009** ↑	−

*Bold values indicate significant differences between muscular contraction obtained with row and column modalities. Up and down arrows are used to specify if the mode represented in the selected row has significantly higher (↑) or lower (↓) muscular contraction than the mode represented in the respective column of the table*.

##### Passive Mode (P)

In *P* mode, the robot entirely performs the movement, and the subjects were asked to simulate the “passive” behavior by relaxing their muscles along the movement, and by not counteracting to residual trajectory errors. As expected, the normalized activation of biceps and triceps was minimal, which confirmed the user's “passive” behavior (Figure 10). Considering the biceps activation during the flexion phase, we found a significant difference (i.e., *p* < 0.05) for all modalities except *W* mode. Instead, triceps contraction during the extension phase resulted in significant difference with *W, R*, and *H* modes (Table 3).

##### Corrective Mode (C)

When in *C* mode, the activation of the biceps was not statistically different with respect to the *T* mode (*p* = 1), while it was different from the others. The triceps activation plot shows no significant muscular activity during movement in favor of gravity. In fact, the triceps iEMG was not significantly different from the *P* mode where all the muscles are relaxed (*p* = 0.055). The *C* mode also demonstrates similarity to the *T* mode, by which the user substantially uses the contribution gravity in the extension phase, and therefore, the triceps activation is almost null.

##### Weight Counterbalance Mode (W)

The trials performed in *W* mode showed that the biceps contracted during the lifting phase, and the triceps during the descending phase. Triceps contraction during the extension phase was slightly higher than in *C* mode, as the user could not exploit the effect of gravity to complete the movement and had to contract the antagonist muscles to counteract the robot weight counterbalance.

##### Transparent Mode (T)

Averagely, the users contracted the biceps during the elbow flexion phase and continued to contract during the elbow extension phase to slow down the downward movement. Since the movement was performed against gravity, the triceps muscle was not significantly activated. We can also observe that both biceps and triceps activation profiles of the *T* mode are substantially similar to *C* mode (Figure 10). This result is confirmed by the iEMG (*p* > 0.05).

##### Resistive Mode (R)

In *R* mode, we can observe high biceps contraction during the elbow flexion phase and triceps contraction during the elbow extension. The activation of the biceps during the elbow flexion was significantly higher than all modalities (*p* < 0.05), except the *H* mode. During the elbow extension phase, we observed triceps contraction significantly greater than all the other training modes.

##### Hypergravity Mode (H)

The *H* mode involved especially the biceps muscles. Indeed, in Figure 10, we can observe a great muscular contraction of the biceps during both elbow flexion and extension. The biceps iEMG index during the elbow flexion phase was significantly different from all modalities (*p* ≤ 0.05), except from the *R* one (*p* = 1), where the users were contracting the biceps to overcome the resistance offered by the robot. During the elbow extension, instead, we observed biceps muscular activation significantly higher than all the other training modalities (*p* ≤ 0.001).

#### 5.2.3. Torque Output Results

Regarding the torque output results presented in Figure 10, the right plots show the torque output generated by the elbow-joint system to the users' arm interface. In *P* mode, the measured torque consisted of the torque generated by the motor to complete the task. Such torque is equal to the inverse-dynamic torque needed to passively move the human-robot system along the desired trajectory, besides residual torques that are not rejected by the torque controller. In *C* mode, the measured torque mainly corresponds to the impedance-based torque employed to correct path deviations. Since the participants were well-performing in the task, the torque variability is limited, and it corresponds to the anti-gravity torque of the elbow test-bed, similar to *T* mode. In *W* mode, the system compensates for arm weight, which varies according to the wearer's characteristics. This explains the greater variability and the greater amplitude of torque profiles. In *T* mode, instead, the robot only compensates its weight, with no trajectory correction. Accordingly, the measured torque profiles show a smaller variance, and the trend goes with the cosine of the joint position, as described in Equation (10). The *R* mode shows that torque trends are inversely proportional to the task velocity, demonstrating a viscous frictional behavior. Finally, a visual inspection shows that in *H* mode, the torque output was opposite to the *P* mode. In fact, the assistance in *P* mode was pushing the arm in the opposite direction with respect to the *H* mode, in which the torque output is aligned with the gravity direction.

## 6. Discussion

The literature proposed several high-level training modalities for effective post-stroke rehabilitation treatment. However, their implementation strongly depends on the developed robotic systems. For example, the Harmony exoskeleton exploits an explicit SEA-based impedance controller (Kim et al., 2017), which is similar to our approach, while other exoskeletons, such as ARMin, use instead implicit impedance controllers to promote rehabilitation exercises (Nef et al., 2007; Khan et al., 2015). However, the generalization of these approaches to a large variety of human-robot interaction modalities, their integration in a unified low-level compliant controller, and the validation of the perceived pHRI through the assessment of the voluntary human effort have not been investigated yet.

We developed a compliant controller based on impedance control and implemented it on a test-bed for the elbow flexion/extension. This study aims at validating the proposed unified control framework through two sets of experiments. We first characterized the controller performances. Then, we evaluated the muscular engagement of healthy subjects by operating the robot in six high-level training modalities.

### 6.1. Actuation and Control Characterization

As a first step, we identified a suitable actuation configuration that could be exploited to create a compliant joint for upper-limb rehabilitation robots. We used actuators along with load-cell feedback to provide high-fidelity torque control. In this way, low-impedance behavior can be achieved, and the robot can behave compliantly with respect to the subject, encouraging residual voluntary movements. On top of this configuration, we proposed a generalized explicit impedance-based control law, which includes positive-feedback terms for friction compensation and arm weight counterbalance. We tested the unified controller performances with an elbow flexion-extension test-bed. The experimental results showed that the developed set-up, combined with the proposed low-level controller, exhibited very low impedance at the joint level, imposing negligible resistive torques (less than 0.3 Nm) on the user's free-motion movements. Notably, since the impedance-based corrective term of the unified controller is superimposed to the *T* control mode, achieving a baseline dynamic transparent behavior was a fundamental step to implement compliant rehabilitation strategies. We can conclude that the inner-loop is expected not to influence the high-level behavior, and it can be considered an ideal torque source.

Due to the developed controller's inner explicit torque feedback control, most of the disturbance torques introduced by the high-ratio gearbox could be reject, without the need for accurate model-based compensation. With these results, we demonstrated that the proposed approach was effective in implementing different virtual stiffness and damping values, that were performed by the robot with good accuracy.

### 6.2. Human-Robot Interaction Modalities Validation

With the developed system, we proposed a set of parameters that could implement various levels of pHRI. Specifically, we combined assistance, correction, and resistance to promote a collaborative controller that implements different high-level training modalities. All the previously presented discrete robot-mediated training strategies can be viewed as different points of a continuum of corrective assistance, counterbalance assistance, and resistance. We underline that this study aims not to define a single set of parameters but to test the hypothesis that the parameter space—if properly explored—can be exploited to move across different rehabilitation scenarios. In particular, we included and tested six rehabilitation modalities, as described in section 3.3.

In this study, we evaluated the capability of the proposed framework to realize a wide range of pHRI by measuring the voluntary muscular activity of healthy subjects in a controlled and replicable experimental protocol. We compared the biceps and triceps muscular activity of 14 healthy subjects under the identified rehabilitation modalities. At the same time, the angular position followed by users and the torque output generated by the elbow-joint system were measured.

The kinematics experimental results demonstrated that the subjects could keep full control of the robotic link while performing elbow flexion/extension tasks. Consequently, the results confirm two crucial hypotheses. First, participants' kinematics performances did not show significant difference across the presented training modalities. Second, all the subjects were able to follow the desired trajectory within the maximum tolerance of about 0.1 radians (about 5.73 degrees). For these reasons, we posit that, under all tested conditions, all subjects could fulfill the required motor tasks in terms of trajectory tracking, range of movement and timing, no matter the level of assistance/resistance provided. Thus, we could compare the electromyographic data across modalities. We observed trajectory tracking to be less accurate than in a position controlled system (especially for the *P* mode). In fact, the impedance control scheme, due to the pure spring-damper correction, introduces bias offset errors to the trajectory tracking control problem that are not negligible. Contrarily, a position control scheme would reject such errors, but it would not provide compliant behavior with the human arm. Furthermore, in applications by which the robot is coupled with a fragile human arm, achieving precise positioning is not a critical aspect, but it is more important to avoid high interaction torques that can be uncomfortable or potentially hazardous to the wearer.

As desired, we observed that the different human-robot interaction modalities implemented with the unified controller induced different muscular activation patterns, both in *biceps* and *triceps brachii*, according to the selected training mode. The interaction modalities ranged from a full robot action with almost null muscular contribution (*P* mode), to training paradigms where the robot resists and challenges the users, requiring them an extra muscular effort to accomplish the task (*R* and *H* modes).

The *T* mode was considered the baseline reference since it describes the behavior by which neither assistance nor resistance is provided to the user during the task. In fact, the muscular effort registered in *T* mode corresponds to the natural free task execution. During elbow-flexion, we observed a medium biceps contraction, while the triceps were characterized by a slight co-contraction. During the extension phase, instead, a modulated contraction of the biceps is used to control the downward motion provided by gravity, while the triceps were again not significantly activated, given that the movement was performed in favor of gravity.

We also observed that both *C* and *W* modes promoted similar biceps contractions that are significantly higher with respect to *P* mode. However, when the weight counterbalance was active (i.e., *W* mode), the triceps experienced greater contraction with respect to the other training modalities. Therefore, these results indicate that such modalities induced the physiological contraction of biceps muscles, and that the controller was inducing slightly greater motor antagonistic activation when weight counterbalance assistance was present. Comparing results obtained in *T* mode with the *C* mode, we could interestingly observe that the activation profiles in the two modalities were comparable, despite the *C* mode allowing a reduced effort and avoid any fail in task execution, providing assistance whether the user is not capable of completing the task or is too slow.

We can also observe that, given that the participants were performing controlled movements (i.e., healthy subjects followed a trajectory pre-defined in position and velocity) with comparable performances, the controller was able to induce muscular patterns in the *C* and *W* modes that are not significantly different from the baseline *T* mode. We can also verify that the torque output in these modalities almost followed the robot weight counterbalance torque (i.e., *T* mode) and that the residual dynamic torque to complete the tasks was generated by users' voluntary contraction. Therefore, we can derive that the proposed control system could correctly implement the assist-as-needed paradigm, helping the user to accomplish the task while inducing the physiological muscular activation pattern.

Instead, in *R* and *H* modes, the statistical analysis confirmed that, for both biceps and triceps, significant greater muscular contraction levels were reached with respect to other modalities. In particular, the *H* mode can be regarded as equivalent to gym-like exercises. In fact, the robot trained the biceps along with the whole movement, during both elbow flexion and extension movements, as if the user was performing the task with payload weights. On the contrary, in *R* mode, the robot trained both muscles during the task: the biceps contracted during the flexion phase, and the triceps during the extension phase.

These results demonstrated that the proposed unified controller could provide low-impedance and high-impedance correction, low-resistance and high-resistance behavior, rendering different levels of pHRI and inducing different levels of muscular contraction and subject's involvement.

### 6.3. Potential Impacts for Neurorehabilitation

From the rehabilitation point of view, the goal is to achieve efficient motor control that should be as similar as possible to the free task scenario, i.e., the *T* mode. Purely corrective strategies (such as *C* mode), around the desired trajectory, modulate the assistance without impacting the muscle recruitment strategy but guaranteeing the completion of the task. Instead, we noticed that in the *W* mode, which involved anti-gravity compensation, the triceps contracted during the extension phase. This implies agonist-antagonist coordination that is entirely different from the natural one, and therefore, it could potentially induce unnatural muscular synergies. From these experimental trials, we observed that anti-gravity compensation of the human arm could induce non-physiological muscular activation, potentially leading to maladaptive plasticity. In this view, a purely corrective approaches might be more effective. However, further investigation is needed to confirm this hypothesis, involving upper arm and forearm tests on the target population.

Finally, the proposed *R* and *H* methods were able to motivate and induce challenging exercises to the subject, training both agonist and antagonist muscles. For this reason, the presented approach could also be applied to the recovery from sports and orthopedic injuries. We claim that the controller could be initially employed to assist the motion during the early stages of the physiotherapy and then—by switching modalities—to improve the muscle mass recovery.

Overall, the controller and the developed hardware confirmed suitability for implementing the training modalities needed for effective physical therapy treatment. With these advancements, we can conclude that the proposed compliant controller might assist the subject along the upper-limb rehabilitation treatment process, from stages when the patient is completely hemiplegic toward the functional recovery of the limb. Future studies will involve the application of this approach to post-stroke patients to assess its efficacy toward motor recovery. Although we developed a compliant joint for the elbow training, future studies can involve the translation of the proposed solution to multi-degrees-of-freedom applications. Indeed, the joint-space control scheme can be replicated for each joint of the robotic chain, and more sophisticated centralized algorithms for arm weight compensation can be implemented.

## 7. Conclusion

In this study, we presented and validated a human-robot cooperative controller for upper-limb robot-mediated neurorehabilitation. The design of the control framework took inspiration from motor learning and neurophysiological aspects, which suggest that good collaboration between the impaired subject and the therapeutic device is preferred to induce effective motor recovery in neurological survivors. In this sense, we found strong evidence that the proposed controller guaranteed dynamic transparency—to promote users' voluntary movements—and produced variable assistance and resistance levels—to tune the rehabilitation treatment according to the subject's performance and involvement.

We demonstrated through electromyographic monitoring that a proper combination of stiffness, damping and weight assistance could properly induce various levels of muscular activation and the subject's participation, namely promoting different human-robot interaction modalities. We believe that since a collaborative controller should provide the minimal amount of assistance to complete the tasks, the presented high-level modalities can be considered as different points of a continuum, and we posit that they can be potentially selectable according to the stage of motor recovery, involving the subject in the completion of the rehabilitation treatment. Our results suggest that the presented collaborative framework is suitable for these purposes. Future studies will extend this approach to multiple degrees of freedom robots and investigate the optimal adaptation control law that makes the controller learn and adapt to the subject's performances in a therapist-like manner. Finally, the efficacy of such a controller on neurological motor recovery will be assessed on post-stroke patients in future studies.

## Data Availability Statement

The raw data supporting the conclusions of this article will be made available by the authors, without undue reservation.

## Ethics Statement

All the experiments that involved human subjects were approved by the Ethical Committee of Politecnico di Milano (Opinion n. 13/2021). All participants provided written informed consent to participate in the study. Written informed consent was obtained from the individual(s) for the publication of any potentially identifiable images or data included in this article.

## Author Contributions

SD, MG, AP, and FB conceived the presented idea. SD developed the theory and implemented control architecture. SD, MG, and VL conceived testing protocols and performed data analysis. SD and VL performed measurements and drafted the manuscript. All authors discussed the results and contributed to the final manuscript and made a significant contribution to the review of the manuscript, read, and approved the final manuscript.

## Funding

We gratefully acknowledge the funding provided by the AGREE project (Regione Lombardia, Pre-commercial procurement, Ref. ARCA_2018_132).

## Conflict of Interest

SD, FB, AP, and MG hold shares in AGADE srl, Milan, Italy. The remaining author declares that the research was conducted in the absence of any commercial or financial relationships that could be construed as a potential conflict of interest.

## Publisher's Note

All claims expressed in this article are solely those of the authors and do not necessarily represent those of their affiliated organizations, or those of the publisher, the editors and the reviewers. Any product that may be evaluated in this article, or claim that may be made by its manufacturer, is not guaranteed or endorsed by the publisher.
